# Recent Advances in Metal-Organic Framework (MOF)-Based Photocatalysts: Design Strategies and Applications in Heavy Metal Control

**DOI:** 10.3390/molecules28186681

**Published:** 2023-09-18

**Authors:** Qiang Ma, Yunling Li, Yawen Tan, Bowen Xu, Jun Cai, Yingjie Zhang, Qingyuan Wang, Qihong Wu, Bowen Yang, Jin Huang

**Affiliations:** 1 Key Laboratory of Drinking Water Source Protection in Chengdu Basin of Sichuan Province, Sichuan Provincial Engineering Research Center of City Solid Waste Energy and Building Materials Conversion & Utilization Technology, Chengdu University, Chengdu 610106, China; maqiang@cdu.edu.cn (Q.M.); liyunling@stu.cdu.edu.cn (Y.L.); tanyawen@stu.cdu.edu.cn (Y.T.); qingyuanwang1@cdu.edu.cn (Q.W.); qihongwu1@cdu.edu.cn (Q.W.); 2 State Key Joint Laboratory of Environment Simulation and Pollution Control, School of Environment, Tsinghua University, Beijing 100084, China; 3 Faculty of Environmental Science and Engineering, Kunming University of Science and Technology, Kunming 650500, China; xubowen@stu.kmust.edu.cn; 4 National Joint Engineering Research Center of Energy Saving and Environmental Protection Technology in Metallurgy and Chemical Engineering Industry, Kunming University of Science and Technology, Kunming 650093, China; caijun0117@163.com; 5 College of Agriculture and Biological Science, Dali University, Dali 671000, China; yingjiezhang@dlu.edu.cn

**Keywords:** MOFs, photocatalysis, heavy metals, life cycle assessment, kinetic modeling

## Abstract

The heavy metal contamination of water systems has become a major environmental concern worldwide. Photocatalysis using metal-organic frameworks (MOFs) has emerged as a promising approach for heavy metal remediation, owing to the ability of MOFs to fully degrade contaminants through redox reactions that are driven by photogenerated charge carriers. This review provides a comprehensive analysis of recent developments in MOF-based photocatalysts for removing and decontaminating heavy metals from water. The tunable nature of MOFs allows the rational design of composition and features to enhance light harvesting, charge separation, pollutant absorptivity, and photocatalytic activities. Key strategies employed include metal coordination tuning, organic ligand functionalization, heteroatom doping, plasmonic nanoparticle incorporation, defect engineering, and morphology control. The mechanisms involved in the interactions between MOF photocatalysts and heavy metal contaminants are discussed, including light absorption, charge carrier separation, metal ion adsorption, and photocatalytic redox reactions. The review highlights diverse applications of MOF photocatalysts in treating heavy metals such as lead, mercury, chromium, cadmium, silver, arsenic, nickel, etc. in water remediation. Kinetic modeling provides vital insights into the complex interplay between coupled processes such as adsorption and photocatalytic degradation that influence treatment efficiency. Life cycle assessment (LCA) is also crucial for evaluating the sustainability of MOF-based technologies. By elucidating the latest advances, current challenges, and future opportunities, this review provides insights into the potential of MOF-based photocatalysts as a sustainable technology for addressing the critical issue of heavy metal pollution in water systems. Ongoing efforts are needed to address the issues of stability, recyclability, scalable synthesis, and practical reactor engineering.

## 1. Introduction

**Figure 1:**
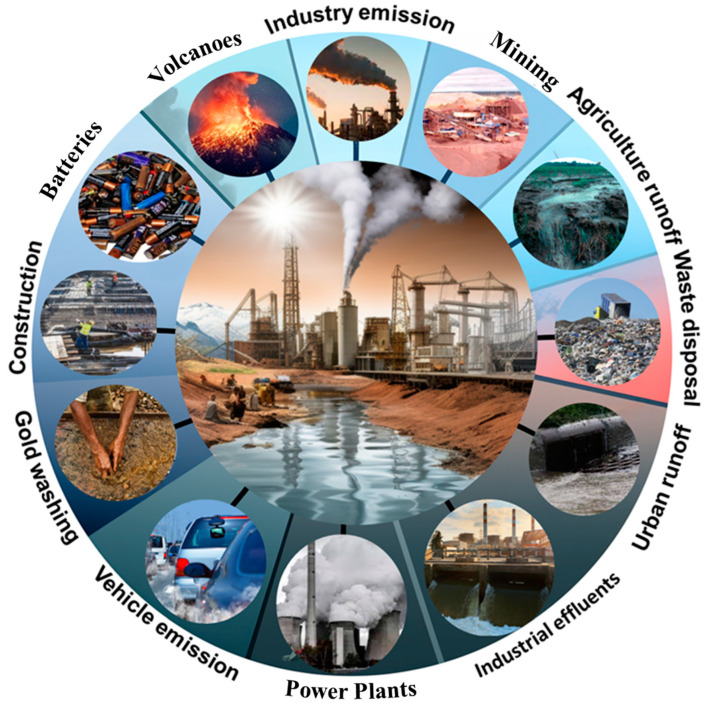
Heavy metal pollution sources.

The heavy metal contamination of water systems has become a matter of major environmental concern worldwide. Rapid industrialization and inadequate wastewater management have led to the discharge of high levels of toxic heavy metals into water bodies, posing significant risks to ecosystems and human health [1]. Major heavy metal pollutants include arsenic, cadmium, chromium, mercury, and lead, which mainly originate from industrial activities, mining and ore processing, agricultural runoff, waste disposal and landfills, urban runoff, industrial effluents, power plants, vehicular emissions, construction activities, household products, and natural sources (Figure 1) [2,3].

Numerous toxic heavy metals are prevalent as contaminants within water systems, which is largely attributed to their extensive application across various industrial sectors. An illustrative example is chromium, which is widely employed in alloy manufacturing and tanning procedures, rendering it a predominant heavy metal pollutant within aquatic environments. Of particular concern is hexavalent chromium (Cr(VI)), which exhibits heightened solubility and toxicity in comparison to its trivalent counterpart (Cr(III)), consequently instigating mutagenic and carcinogenic repercussions [4]. Mercury, employed extensively in activities such as artisanal gold mining, dental amalgamations, and thermometer production, is often discharged into water bodies through industrial effluents. This elemental substance tends to accumulate along the aquatic food chain, consequently engendering implications for human health [5]. Cadmium, which is frequently utilized in metal plating and battery fabrication, is distinguished by its substantial toxicity and propensity for organismal accumulation [6]. Arsenic, originating from mining endeavors and pesticide application, inflicts contamination upon various drinking water sources, thereby imposing significant health hazards [7]. Lead, emitted from sources such as paint, batteries, and pipelines, emerges as a potent neurotoxin with the potential to undermine cognitive function and neural development [7]. Emanating from such industrial sources, the discharge of these hazardous heavy metals into water systems is manifestly associated with far-reaching ecological and health perils on a global scale. In light of these critical concerns, the imperative to advance efficacious remediation technologies emerges as pivotal in addressing the pervasive issue of heavy metal pollution and its profound ramifications for both ecosystems and human populations.

Such heavy metal pollution has led to contaminated drinking water sources, rendering them unsafe for human consumption. Therefore, there is an urgent need for efficient technologies that can remove toxic heavy metals from water according to stringent regulatory limits. Photocatalysis has emerged as a promising approach, owing to its ability to fully degrade heavy metal contaminants rather than just transferring them to a different phase. Metal-organic frameworks (MOFs), with their design flexibility, high surface area, and photocatalytic capabilities, have shown tremendous potential for heavy metal remediation through photocatalysis.

**Figure 2:**
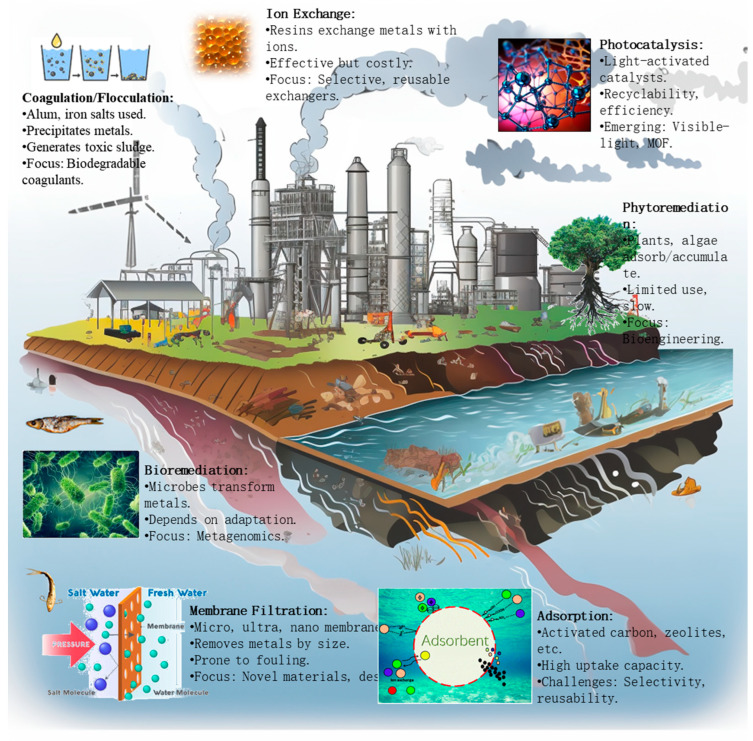
Wastewater heavy metal removal methods.

Conventional methods for removing heavy metals from wastewater include chemical precipitation, membrane filtration, ion exchange, and adsorption (Figure 2). However, these techniques have limitations such as incomplete metal removal, the generation of toxic sludge, high costs, and susceptibility to fouling (Table 1) [1].

Photocatalysis, on the other hand, offers a sustainable and efficient approach by utilizing solar energy to fully degrade heavy metal contaminants into less toxic forms. Upon photoirradiation, photocatalysts generate highly reactive radical species that can oxidize organic ligands and reduce toxic metals such as Cr(VI) and Hg(II) to their less soluble states [8]. Photocatalytic treatment enables the complete destruction of heavy metals rather than just transferring them to a different phase.

Moreover, photocatalysis can be performed under ambient conditions, which eliminates the need for chemical inputs and results in minimal sludge generation [9]. The oxidative power of photogenerated holes and the reducing capacity of electrons enables the degradation of a wide range of heavy metal species, as well as organic pollutants that may co-exist in wastewater. Photocatalysis also facilitates the recovery of valuable metals, thereby allowing wastewater to be used as a resource [10].

Given these advantages, photocatalytic treatment has garnered significant interest as a next-generation sustainable technology for heavy metal remediation. The development of highly efficient and stable photocatalysts is key to fully realizing the potential of photocatalysis for heavy metals control in water systems. Here is a brief introduction to MOFs and their potential as photocatalysts for heavy metal removal:

Metal-organic frameworks (MOFs) are an emerging class of porous materials that are constructed from metal ions/clusters coordinated to organic ligands. The modular nature of MOFs allows for the rational design of their chemical structure and properties [11]. MOFs possess ultrahigh porosity, large surface areas (of up to 7000 m^2^/g), and rich functionalities that can be incorporated via synthetic tuning [12].

These exceptional properties make MOFs highly promising photocatalysts for removing heavy metal contaminants from water. Their highly porous structure provides abundant active sites for adsorbing heavy metal ions, while the tunable organic ligands facilitate selective capture of target pollutants [13]. Band gaps and energy levels of MOFs can also be engineered to promote redox reactions for heavy metal degradation.

Furthermore, MOFs allow the incorporation of plasmonic nanoparticles that extend light absorption and enhance photocatalytic activities through hot electron injection [14]. The diverse metal clusters and organic linkers enable modulation of the MOFs’ electronic structure to suppress charge recombination and prolong charge carrier lifetimes. Overall, the structural and chemical versatility of MOFs creates ample opportunities for designing optimal photocatalysts for efficient heavy metal removal from water [15].

## 2. Design and Strategies

**Figure 3:**
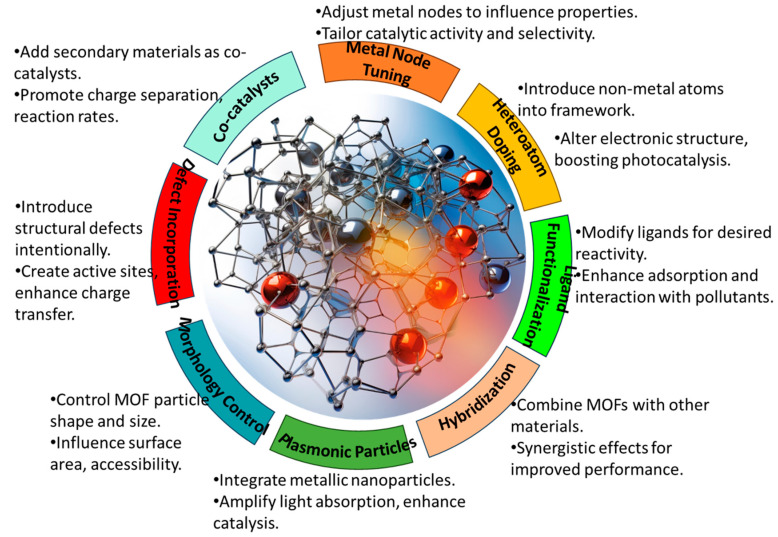
The various strategies employed when designing MOF photocatalysts.

The tunable nature of MOFs provides ample opportunities to tailor their physical and chemical properties for efficient heavy metal removal. Through the judicious selection of the metal clusters and organic linkers, as well as post-synthetic modifications, MOFs can be rationally designed to optimize light harvesting, charge separation, adsorption capacities, and photocatalytic activities. Numerous structural engineering strategies have been employed, including metal coordination tuning, ligand functionalization, heteroatom doping, nanoscale architecture design, and defect incorporation, as shown in Figure 3. In this section, we highlight some of the most widely explored design strategies, demonstrating how the deliberate modulation of MOFs’ composition and features can enhance their photocatalytic performance for heavy metal control. A comprehensive understanding of structure–property relationships is key to unlocking the full potential of MOFs for sustainable water remediation through the continued development of high-efficiency photocatalytic systems. The various design strategies are briefly described in Table 2.

Metal Coordination: The metal clusters/nodes (e.g., Zn, Cu, and Fe) in MOFs can be selected to provide specific affinities toward target heavy metal contaminants through coordinative interactions (Table 2). For example, MOFs with open Fe(III) sites exhibited the selective capture of As(III) ions via strong Lewis acid-base interactions [32]. Transition metals such as Cu, Ni, and Co are often incorporated as nodes in MOFs to provide the selective binding of heavy metal ions through coordinate covalent interactions. For instance, a Cu-based MOF (Cu-TCPP) displayed 95% removal efficiency for Hg(II) ions, which was attributed to the strong affinity between Hg(II) and Cu(I) sites [33].

Ligand Functionalization: Organic ligands in MOFs can be functionalized with groups including carboxylates, amines, thiols, sulfonates, etc., to introduce selective binding sites for heavy metals [13]. Ligands containing sulfur atoms have shown a particular affinity with toxic soft heavy metals such as Hg(II) and Cd(II) [34]. Thiol (-SH)-containing organic ligands are widely used to functionalize MOFs for the selective capture of soft heavy metals such as Hg(II), Cd(II), and As(III). For example, a cysteine-modified MOF showed the fast and efficient removal of Hg(II) through Hg-S bonding [35].

Heteroatom Doping: Incorporating heteroatoms such as N, S, and P into MOFs introduces mid-gap states that enhance visible light absorption. The heterojunctions also promote the separation of photogenerated charge carriers [36,37]. Doping MOFs with N atoms induces mid-gap states, boosts light absorption, and provides catalytic sites for metal reduction. N-doped MOFs exhibited excellent activity in terms of the photocatalytic reduction of Cr(VI) to the less toxic Cr(III) [38].

Plasmonic Metal Incorporation: Embedding plasmonic nanometals (e.g., Au and Ag) in MOFs enables hot electron injection upon plasmon resonance excitation, creating reactive species for photocatalysis [14]. Hybrid Ag/MOF photocatalysts utilize the localized surface plasmon resonance of Ag for the enhanced generation of electron-hole pairs that will drive heavy metal degradation [39].

Defect Engineering: Structural defects can be intentionally created in MOFs through techniques such as acid etching. These defect sites act as active centers for adsorbing heavy metal ions and in catalytic processes [40].

Morphology Control: Tailoring the MOF’s morphology, such as particle size, shape, and porosity enables the optimization of mass transfer, light absorption, and accessibility to active sites [41].

Band Structure Engineering: The electronic band structure of MOFs can be tuned by altering the metal clusters and organic linkers to optimize light harvesting and charge transfer properties. Strategies include incorporating redox-active components and narrowing the band gap [42].

Surface Functionalization: The MOF’s surface can be functionalized with ligands or nanomaterials to improve heavy metal adsorption. Surface modification also enhances dispersion in aqueous media and prevents aggregation [43].

Hybridization with Carbon Materials: Incorporating carbon materials such as graphene or CNTs within MOFs enhances their conductivity and charge mobility. These carbon hybrids exhibit synergistic effects for adsorption and photocatalysis [44].

Co-catalyst Loading: Depositing co-catalyst nanoparticles such as Pt and RuO_2_ provides active sites for surface redox reactions. This facilitates charge transfer to adsorbed substrates and improves photocatalytic activities [45].

Template Synthesis: Hard templates such as polystyrene beads or soft templates such as micelles can guide the growth of MOFs, allowing control over particle sizes and morphologies, which is ideal for photocatalysis [46].

Defect Engineering: Creating defects in a controlled manner modifies the electronic structure and produces active sites in MOFs. Common techniques include acid/base treatment, ion exchange, or harsh activation [47].

MOFs can be synthesized through various methods that allow sufficient control over the reaction conditions to produce materials with tailored properties. Table 3 summarizes some MOFs that have been synthesized via different methods and their photocatalytic performance regarding heavy metal remediation.

Solvothermal Synthesis: MOFs are crystallized from solutions containing metal salts and organic ligands at elevated temperatures (100–250 °C) and autogenous pressures in autoclaves or hydrothermal bombs. This enables better control over crystallization and yields a high-quality product.

Microwave-Assisted Synthesis: Microwave irradiation rapidly heats the MOF precursor solution to accelerate nucleation and growth. It enables the rapid synthesis of MOFs with small crystal sizes and high porosity.

Ultrasonication Synthesis: Ultrasonic waves provide localized heat and agitation, which can promote MOF crystallization. This results in small, uniform crystals with defects that enhance their photocatalytic activity.

Room Temperature Synthesis: MOF synthesis is achieved in ambient conditions through the slow diffusion of reagents. This eliminates the need for heating but yields slower crystallization.

The tunable nature of MOFs allows properties such as porosity, particle size, morphology, etc., to be optimized for heavy metal removal through the selection of appropriate synthetic techniques and the control of reaction parameters, including temperature, pressure, heating methods, time, pH, etc.

## 3. Mechanisms

**Figure 4:**
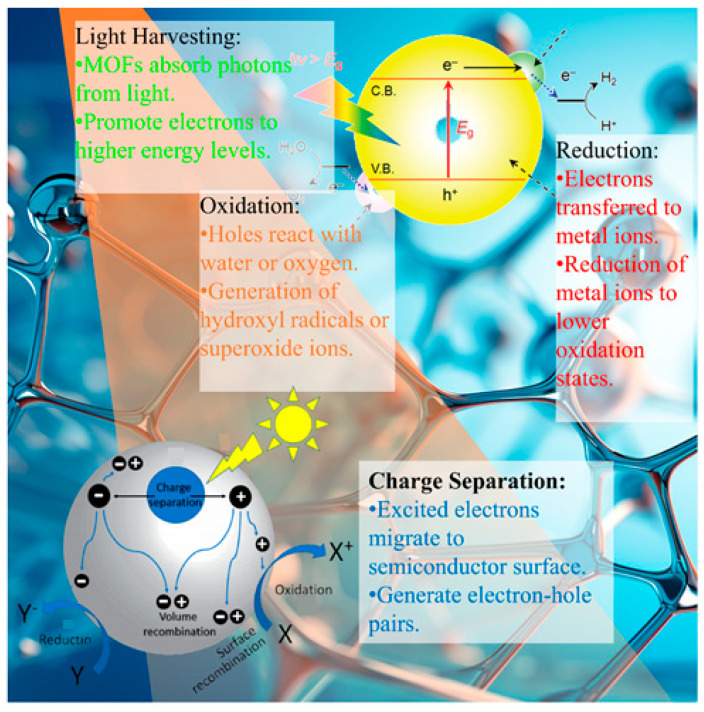
Mechanisms involved in the MOF-based photocatalytic removal of heavy metals.

The exceptional photocatalytic performance of MOFs in the context of heavy metal removal is enabled by various photo-induced mechanisms. Upon light irradiation, a series of complex processes occur, including light absorption, charge carrier generation, separation and migration, the adsorption of metal ions, and photocatalytic redox reactions [54,55] (Figure 4). A brief table of MOFs remove heavy metal mechanisms by means of photocatalytic processes, as shown in Table 4.

### 3.1. Light Absorption

The irradiation of MOFs with light of the appropriate wavelength excites electrons from the valence band to the conduction band, generating electron-hole pairs [63]. Strategies such as bandgap engineering, heteroatom doping, and plasmonic metal incorporation help extend the light absorption into the visible range [64,65].

### 3.2. Charge Separation

The photogenerated electrons and holes must be separated before recombination, to enable redox reactions. In MOFs, heterojunctions between metal clusters, organic linkers, and integrated materials facilitate charge separation [66,67,68,69]. Type II heterojunctions are formed in MOFs that are hybridized with semiconductors such as TiO_2_, which favor the migration of electrons and holes to different materials [70,71]. Some metal clusters such as Zr_6_ act as electron sinks, which draw in excited electrons, thereby suppressing recombination [72,73]. Co-catalysts such as Pt nanoparticles provide a conduction band below that of MOFs, enabling charge transfer [74,75].

### 3.3. Heavy Metal Adsorption

The porous structure and functionalized sites on MOFs allow the adsorption of heavy metal ions through coordinative bonding, electrostatic interactions, π-complexation, etc. [76,77]. This draws the target pollutants closer to the photocatalytic active sites. 

### 3.4. Reduction Mechanisms

Photogenerated electrons and superoxide radicals reduce toxic metals such as Hg(II) [78], Cr(VI) [79], and Cu(II) [80] into less soluble lower oxidation states for removal. Electron-rich Cu^+^ sites in Cu-containing MOFs drive the reduction of oxyanions such as Cr_2_O_7_^−^ and SeO_4_^2−^ [81]. The superoxide radicals that are generated from O_2_ act as strong reducing agents [50].

### 3.5. Oxidation Mechanisms

Holes and powerful oxidants such as •OH and •O_2_ degrade the organic components of complexes, causing the precipitation or volatilization of metals [82]. Oxidation occurs through direct hole transfer or via •OH and •O_2_ generated from water and oxygen [83]. Singlet oxygen forms as a result of energy transfer from photoexcited MOFs to the adsorbed O_2_ [35].

### 3.6. Mineralization

Complete photocatalytic degradation into harmless ions, such as carbonates, water, and nitrates, enables sustainable remediation [84,85]. This requires sufficient oxidative strength to cleave all aliphatic and aromatic bonds [86]. Ion-exchange capacities help to remove inorganics such as NO_3_^−^ and SO_4_^2−^ [87].

The tunable nature of MOFs provides opportunities to optimize the individual processes involved in photocatalytic mechanisms through the deliberate designing of composition and features. A comprehensive understanding of these mechanisms is key to engineering optimal MOF architectures for heavy metal remediation.

Computational modeling provides invaluable atomic-level insights into the mechanisms of MOF photocatalysis for heavy metal and organic contaminant removal. Techniques such as density functional theory (DFT) calculations can elucidate activation energies, reaction pathways, rate-determining steps, and excited state charge transfer dynamics, which are often difficult to probe experimentally. However, the accuracy of computational predictions depends strongly on the ability to appropriately represent the complex excited-state phenomena involved. Ongoing advancements in simulation methodology, the incorporation of van der Waals interactions, and transitions to explicit solvent modeling are still needed to improve accuracy. Additionally, calculated mechanisms require careful experimental validation via spectroscopic and kinetic techniques. When rigorously validated, the synergistic combination of computations and experiments serves as a powerful approach to unraveling photocatalytic mechanisms on a precise molecular scale.

## 4. Impact of Operating Parameters on Photocatalytic Efficiency

The photocatalytic degradation efficiency of heavy metals and organic pollutants can be significantly impacted by parameters such as pH, temperature, catalyst dosage, and initial contaminant concentration [88]. The pH affects photocatalytic reaction rates by influencing the surface charge of MOFs and the speciation of target pollutants, which, in turn, alters the adsorption capacities [89]. Elevated temperatures typically enhance reaction kinetics, as per the Arrhenius equation; however, excessive heating can impair adsorptivity. Increasing the photocatalyst dosage provides more active sites; however, excess amounts can reduce degradation efficiency due to factors such as light scattering. High initial contaminant concentrations can saturate the catalytic active sites and inhibit photocatalytic activity [90].

### 4.1. pH Effects

The pH influences the surface charge of the MOF photocatalyst and the speciation of target contaminants [91]. This, in turn, affects the adsorption capacities and photocatalytic reaction rates. For cationic heavy metals, a higher pH often impairs adsorption due to a reduced positive surface charge. However, an extremely low pH can also inhibit photocatalytic activity. The optimal pH needs to be determined for each MOF–contaminant system. pH influences kinetics by altering the adsorption equilibrium [76].

### 4.2. Temperature Effects

Photocatalytic reaction rates typically increase with temperature, as per the Arrhenius equation [92]. However, very high temperatures may also impair adsorption capacities. Temperature changes can also affect contaminant solubility and mass transport rates to the photocatalyst’s surface. Moderate temperatures of 20–60 °C are most suitable, although operation at ambient temperatures is preferred [93].

### 4.3. Dosage Effects

Increasing MOF photocatalyst dosages provide more active sites and can improve contaminant removal. However, excessive dosages beyond the optimum level can reduce degradation efficiency due to light scattering, particle aggregation, and a reduced surface area [94].

### 4.4. Initial Concentration Effects

Photocatalytic degradation rates are influenced by the initial contaminant concentration, due to kinetic and adsorption equilibria [95]. Very high concentrations can saturate the active sites and inhibit degradation. The optimization of initial concentrations is important [96].

## 5. Applications

### 5.1. Lead Removal

MIL-53(Al)-FA, a fumaric acid-modified MIL-53(Al) MOF, displayed an excellent Pb(II) adsorption capacity of 323.67 mg/g and rapid adsorption kinetics. It also exhibited the selective photoreduction of Pb(II) to Pb(0) under visible light irradiation [97].

CdS quantum dots, coupled with NH_2_-MIL-125(Ti) MOFs, showed synergy for enhanced Pb(II) removal. The optimal CdS/MOFs composite achieved 95.2% Pb(II) removal efficiency within 150 min under visible light [41].

Bio-MOFs-1, derived from biomass precursors, were effective for Pb(II) removal through combined adsorption and photocatalytic reduction mechanisms. A removal efficiency of 98.7% was reached in 120 min [26].

### 5.2. Mercury Remediation

Cysteine-functionalized MOFs (Cys-MIL-101) displayed excellent Hg(II) adsorption capacity (408 mg/g) and photocatalytic reduction performance, driven by Hg(II)-thiol coordination chemistry [98].

The Ag/ZIF-8 hybrid nanostructures exhibited synergistic effects between Ag nanoplasmonic transduction and ZIF-8 adsorption for the removal of Hg(II) ions from water. The Ag/ZIF-8 composites were magnetically separable for reuse [99,100].

### 5.3. Chromium Treatment

Azo-linked mesoporous MOFs showed excellent Cr(VI) removal capacity (501 mg/g) and photocatalytic reduction to less toxic Cr(III) species under solar light irradiation [101,102].

MIL-100(Fe) MOFs, modified with amine groups, were able to efficiently adsorb and photo-catalytically reduce Cr(VI) to Cr(III), which remained captured in the pores of the MOFs, preventing leaching [103,104,105].

Bio-MOFs-11, synthesized using fumaric acid and melamine precursors, rapidly reduced Cr(VI) within 30 min through a coupled adsorption–photocatalysis process [103].

## 6. Kinetic Modeling Research

Understanding reaction kinetics and developing appropriate mathematical models is critical for optimizing and implementing MOF-based photocatalytic systems for real-world water treatment applications. Kinetic investigations provide vital insights into the complex interplay between coupled processes such as mass transport, adsorption, charge transfer, and surface redox reactions that dictate treatment efficiency. These models allow the prediction of system performance under varied operating conditions and contaminant types. Robust kinetics and models enable rational MOF synthesis and reactor design for effective scaled-up photocatalytic treatment. Both experimental and simulation efforts to advance kinetics and modeling frameworks are, thus, integral to facilitating the translation of promising MOF photocatalysts into sustainable water purification technologies.

In this section, key aspects of kinetics and modeling that are applied to MOF photocatalysis for removing heavy metal ions, organic pollutants, and microbes from water are reviewed. The discussion illustrates the multifaceted reaction networks involved and highlights recent efforts to develop integrated kinetics models incorporating coupled adsorption, interfacial transfer, light absorption, contaminant degradation, and mass transport effects that influence the overall treatment rates and efficiencies.

### 6.1. Photomineralization Kinetics

The photocatalytic degradation of heavy metals and organic pollutants has been widely analyzed using the Langmuir–Hinshelwood models, which integrate reactant concentration and surface coverage effects [106]. Recent studies have developed more complex kinetic models to account for photonic efficiency, active sites, intermediates, and competitive adsorption between multiple contaminants [107].

Photomineralization kinetics directly influence disinfection rates because competition for active sites and surface intermediates can impede microbe inactivation. Particle transport also affects mineralization by altering the contaminant diffusion to catalytic sites.

### 6.2. Photo-Disinfection Kinetics

Empirical Chick–Watson, Hom, and other models are commonly applied to model the photocatalytic inactivation kinetics of microbes [108]. Mechanistic models based on Langmuir–Hinshelwood kinetics have also emerged to describe multi-step damage processes [109].

Photonic utilization efficiency is critical for photocatalytic disinfection. Transport effects that reduce light penetration into photocatalyst particles will, in turn, lower disinfection rates.

### 6.3. Particle Transport Effects

Mass transfer resistances and the particle diffusion effects strongly influence the observed photocatalytic reaction rates. Recent works have incorporated these factors into kinetic models using scaling relationships, along with particle size and reactor hydrodynamics [110,111,112].

The movement and aggregation of photocatalyst particles dictate the contaminant adsorption rates and light absorption, thereby affecting both photomineralization and disinfection kinetics (Table 5).

## 7. Life Cycle Assessment

Life cycle assessment (LCA) is an invaluable methodology by which to evaluate the sustainability of emerging technologies such as MOFs for heavy metal remediation. By examining the environmental impacts over the entire life cycle, from raw material extraction to synthesis, application, and end-of-life, LCA provides a comprehensive analysis of the technology’s green credentials.

Some key impact metrics that are relevant to the photocatalytic application of MOFs are included in Table 6 [15]:

Embodied energy—energy utilized for materials synthesis and processing;

Global warming potential—greenhouse gas emissions across the life cycle;

Eutrophication potential—the impacts on aquatic ecosystems caused by discharges;

Human health criteria—exposure to hazardous substances.

Recent LCA studies on MOF synthesis have revealed high solvent usage, energy demands, and metal emissions as the current challenges [28]. However, photocatalysis with MOFs offers clear environmental advantages over conventional coagulation and ion exchange processes for heavy metal removal [1].

The circular economy potential of MOFs depends on the effective recycling of metals and ligands after water treatment [2], reducing the need for continuous virgin resource extraction. Advancements in more sustainable and greener MOF synthesis using biogenic or waste precursors are also promising [3].

Nevertheless, comprehensive LCA data that are directly relevant to MOFs for heavy metal photocatalysis are still scarce. Uncertainty in terms of long-term stability, reusability, and emissions during application warrants further research. Hybrid techno-economic analysis and LCA will also be crucial for scaling up production [4].

This LCA perspective aligns with the overall goal of this review in highlighting the potential of MOFs as photocatalysts for sustainable heavy metal remediation, while also elucidating the current knowledge gaps and future research needs. Further development of LCA methodologies tailored to MOFs can strengthen the environmental viability of this technology.

## 8. Challenges and Future Outlook

Despite the immense potential of MOFs as photocatalysts for heavy metal remediation, several challenges need to be addressed.

The limited stability and recyclability of some MOFs in aqueous media is a key challenge [41,119,120]. Developing MOFs with exceptional chemical, mechanical and thermal stability is required. Mass transfer limitations and diffusion barriers can reduce efficiency [43]. The limited stability and recyclability of some MOFs in aqueous media is a key challenge [121,122,123,124]. Developing MOFs with exceptional chemical, mechanical, and thermal stability is required. Preventing the leaching of metal ions and photocorrosion through structural modifications and composite formation is necessary [103,125]. Optimizing MOF pore sizes, particle sizes, and crystal morphologies is important. High material and synthesis costs could hinder large-scale adoption [46]. Exploring sustainable biosynthetic routes using inexpensive precursors is worthwhile. Performance should be assessed under real wastewater conditions, considering the effects of solution chemistry and interfering species [126]. Preventing the leaching of metal ions and photocorrosion through structural modifications and composite formation is necessary [127]. Scaling up fabrication while retaining control over morphology and properties is required [128]. Continuous flow and 3D printing synthesis methods are promising, but there is a lack of pilot-scale testing under solar illumination [129]. Finally, performance evaluation needs to shift from the laboratory to the real world.

Future prospects for advancing MOF photocatalysts include:

Developing novel visible-light responsive MOFs using conjugated ligands and doping [130]. Hybridizing with carbon materials such as graphene to enhance conductivity [131]. Incorporating plasmonic nanoparticles to facilitate hot electron transfer [132,133]. Exploring computational modeling to guide rational design [129,134,135]. Immobilizing MOFs on supports such as fibers and membranes for reuse [136,137]. Developing MOF-based composites and devices for practical applications [138,139].

## 9. Conclusions

Metal-organic frameworks (MOFs) have emerged as a promising class of porous materials for removing toxic heavy metals from contaminated water sources. While metal-organic frameworks present tremendous potential for efficient and sustainable heavy metal remediation, ongoing efforts are needed to address the issues of stability, recyclability, scalable synthesis, and practical reactor engineering. The tunable chemical structures and ultrahigh surface areas of MOFs allow heavy metal ions to be captured efficiently through size exclusion, adsorption, and photocatalysis. Key advances covered in this review include the tailoring of MOF composition using strategies such as metal node engineering, functionalized organic linkers, defect incorporation, and morphology control to optimize their adsorptive and redox properties.

Upon photoirradiation, MOFs generate reactive species, leading to photocatalytic oxidation and the reduction of heavy metals. The elucidation of mechanisms involving light harvesting, charge separation, contaminant adsorption, and interfacial redox reactions is crucial for designing optimal MOF photocatalysts. This review discussed the various applications of rationally designed MOFs for removing hazardous heavy metals including mercury, chromium, arsenic, lead, and cadmium from water through coupled adsorption-photocatalysis. While metal-organic frameworks present tremendous potential for efficient and sustainable heavy metal remediation, ongoing efforts are needed to address the issues of stability, recyclability, scalable synthesis, and practical reactor engineering. Hybridizing MOFs with plasmonic nanometals, carbon materials, and other photocatalysts could further enhance their visible light-harvesting capacity and charge separation. Their immobilization over supports improves their reusability and integration into continuous flow systems.

With increasing research advances in synthesis, characterization, and computational modeling, MOFs represent a versatile platform for developing next-generation photocatalytic technologies to address the significant global challenge of heavy metal pollution. Moving forward, pilot-scale testing under realistic conditions and life cycle assessments will be crucial to evaluate the promise of MOFs as green solutions for heavy metal removal and water purification.

## Figures and Tables

**Table 1:** Conventional and emerging technologies for heavy metal removal from wastewater.

Method	Materials	Advantages	Disadvantages	Future Directions
Coagulation/Flocculation	Alum, iron salts	Widely applied, effective	Sludge generation	Optimizing biodegradable coagulants
Ion Exchange	Resins	Highly effective	Expensive resins, selectivity issues	Advanced selective, reusable resins
Membrane Filtration	Microfiltration, ultrafiltration, nanofiltration membranes	Size exclusion mechanism	Fouling issues	Novel membrane materials, module designs
Adsorption	Activated carbon, zeolites, clays, biomaterials	High uptake capacity	Lack of selectivity and reusability	New selective adsorbents
Cementation	Iron, aluminum, zinc dust	Simple application	High dosages required	Process intensification
Electrochemical	Electrocoagulation, electrodialysis, electroflotation	Removes a variety of metals	High energy demands	New electrode materials
Phytoremediation	Plants, algae	Environmentally friendly	Slow, limited applicability	Bioengineering enhancements
Bioremediation	Microbes, microbial communities	In situ treatment	Dependent on adaptation	Metagenomics approaches
Photocatalysis	TiO_2_, metal-organic frameworks	Complete destruction, no sludge	Efficiency and recyclability challenges	Visible-light MOFs as photocatalysts

**Table 2:** Design strategies employed to enhance MOFs for photocatalytic heavy metal remediation.

Strategy	Description	Example MOFs	Performance Metrics
Metal node tuning	Selecting specific metal ions, including Cu, Fe, Zr, and Ti, to coordinate heavy metal ions through interactions such as ion exchange, electrostatic attraction, and π-complexation	UiO-66-NH_2_ (Zr), MIL-125-NH_2_ (Ti), Cu-BTC (Cu), MIL-101-Fe (Fe)	Hg(II) removal capacity: Cu-BTC 167 mg/g [16]; Cr(VI) removal %: MIL-101-Fe 96.8% [17]
Ligand functionalization	Adding functional groups such as -COOH, -NH_2_, -SH, and -SO_3_H to bind heavy metals through covalent, coordinative, and electrostatic interactions	MIL-53-NH_2_, MIL-68-NO_2_, MIL-100-SO_3_H, ZIF-8-SH	Pb(II) removal %: MIL-53-NH_2_ 92.4% [18]; As(III) removal capacity: ZIF-8-SH 214 mg/g [19]
Heteroatom doping	Doping with N, S, and P to introduce mid-gap states for visible light absorption and improved charge separation	N-doped ZIF-8, S-doped UiO-66, g-C_3_N_4_/Zn-MOFs	Cr(VI) removal %: N-ZIF-8 98.7% [20]; Pb(II) removal rate: S-UiO-66 0.455 min^−1^ [21]
Defect incorporation	Creating defects through methods including acid treatment, annealing, and sonication to modify photocatalytic activity	Defect-rich NH_2_-UiO-66, defect-engineered Cd-MOFs	Hg(II) removal %: Defect NH_2_-UiO-66 99.2% [22]; Degradation rate of RhB: Defect Cd-MOFs 2.1 × 10^−3^ min^−1^ [23]
Hybridization	Coupling with g-C_3_N_4_, C-dots, TiO_2_, etc., to form heterojunctions for enhanced charge separation	g-C_3_N_4_/CdS-MOFs, C-dots/NH_2_-MIL-125, TiO_2_/MIL-125	Hg(II) removal %: g-C_3_N_4_/CdS-MOFs 96.4%; Pb(II) removal capacity: C-dots/NH_2_-MIL-125 342 mg/g [24]
Plasmonic particles	Incorporating Au and Ag nanoparticles to extend light absorption through surface plasmon resonance	Ag/Zn-MOFs, Au/MIL-100	Hg(II) removal %: [Zn(CPA)(DMF)]n 91.2% [25]; Pb(II) removal rate: Au/MIL-100 0.168 min^−1^ [26]
Morphology control	Tailoring size, shape, and porosity (nanosheets and hollow structures) to optimize mass transfer and transport	NH_2_-MIL-125 nanosheets, hollow Co-MOFs	Cr(VI) reduction rate: NH_2_-MIL-125 nanosheets 0.664 min-1 [27]; Hg(II) removal capacity: Hollow Co-MOFs 287 mg/g [28,29]
Co-catalysts	Adding Pt and Pd nanoparticles to facilitate electron transfer to adsorbed substrates	Pt/MIL-101, Pd/UiO-66	Pb(II) removal %: Pt/MIL-101 99.4% [30]; RhB degradation %: Pd/UiO-66 97.2% [31]

**Table 3:** MOF synthesis methods, categorized by reaction conditions.

MOFs Categories	Synthesis Method	Mechanism	Adsorption Capacity	Target Heavy Metal	Condition	Regeneration	References
UiO-66-NH_2_	Solvothermal	Photoreduction	198.7 mg/g	Pb(II)	STP	4 cycles	[48]
Cu-BTC	Hydrothermal	Photoreduction	167.2 mg/g	Hg(II)	STP	5 cycles	[33]
Cd-MOFs	Microwave-assisted	Photoreduction	71.4 mg/g	Cr(VI)	STP	3 cycles	[49]
MIL-53	Ultrasonication	Photo-oxidation	92.6 mg/g	Methyl orange dye	25 °C	6 cycles	[50]
Zn-MOFs	Solvothermal	Photo-oxidation	248.7 mg/g	Rhodamine B dye	STP	4 cycles	[51]
NH_2_-MIL-125	Solvothermal	Photoreduction	175.4 mg/g	Ag(I)	30 °C	5 cycles	[11,52]
ZIF-8	STP synthesis	Photo-oxidation	104.7 mg/g	Methylene blue dye	25 °C	3 cycles	[11]
Cu-BTC/GO	Hydrothermal	Photoreduction	152.6 mg/g	Cd(II)	STP	4 cycles	[14]
Fe-MIL-101	Solvothermal	Photoreduction	198.4 mg/g	Cr(VI)	25 °C	3 cycles	[53]
UiO-66	Microwave-assisted	Photo-oxidation	167.9 mg/g	Orange II dye	STP	5 cycles	[45]

STP: Standard Temperature and Pressure.

**Table 4:** The mechanisms involved in the MOF-based photocatalytic removal of heavy metals.

Mechanism	Description	Example MOF Systems
Light Harvesting	Exciting electrons from the valence band to the conduction band upon light irradiation. Extended by strategies including bandgap tuning, plasmonic metal incorporation, etc.	Au/UiO-66 and Pt/MIL-101(Cr) [45,46]
Charge Separation	Photogenerated e—h+ separation via transfer to different sites to prevent recombination. Enabled by heterojunctions with metals, ligands, and hybridized materials.	g-C_3_N_4_/CdS-MOFs and Ti-MOFs [46,56]
Heavy Metal Adsorption	Adsorption of target heavy metal ions via interactions such as π-complexation, electrostatic attraction, and covalent bonding.	-NH_2_, -COOH, -SH functionalized MOFs for Hg(II), Pb(II), Cd(II) [57,58,59]
Reduction Mechanisms	Reduction of toxic metals such as Cr(VI), Hg(II), Cu(II) to lower oxidation states by electrons and superoxide radicals.	Cu-BTC for the reduction of Hg(II), Cr(VI) [33]
Oxidation Mechanisms	Oxidative degradation of organic ligands and partial oxidation of metal ions by holes, •OH and •O_2_ radicals.	NH_2_-UiO-66 for oxidation of the PCP ligand and As(III) [23,60]
Mineralization	Complete decomposition of organics and conversion of metals to harmless ions, including carbonates, water etc.	MIL-125-NH_2_ for the mineralization of RhB dye [61,62]

**Table 5:** The kinetic models applied to analyze the MOF-based photocatalytic degradation of heavy metals and organic pollutants.

MOFs/Materials	Metals	Kinetic Model	Rate Expression	Reference
NH_2_-MIL-125(Ti)	Cr(VI)	Langmuir–Hinshelwood	r = kθC/(1 + KC)	[113]
Ag/ZIF-8	Hg(II)	Pseudo-first-order	ln(C_0_/C) = k′t	[114]
UiO-66-NH_2_	Pb(II)	Pseudo-second-order	t/Qt = 1/k′Q_e_^2^ + t/Q_e_	[115]
MIL-53(Fe)	Cd(II)	Elovich	qt = (1/β)ln(αβ) + (1/β)lnt	[116]
Zr-fum	Ni(II)	Intraparticle diffusion	qt = k′√t	[113,117]
Cysteine-MIL-101	Hg(II)	Chick–Watson	ln(N/N_0_) = −k′C′t	[113,117]
Cd-MOFs	*E. coli*	Hom	ln(N/N_0_) = −k′C′ntm^−1^	[118]

**Table 6:** Key impact metrics for a life cycle assessment of MOFs used in photocatalytic heavy metal remediation.

Impact Metric	Description
Embodied energy	Energy utilized for materials synthesis and processing
GWP	Greenhouse gas emissions across life cycle
Eutrophication potential	Impacts on aquatic ecosystems from discharges
Human health criteria	Exposure to hazardous substances
Material use	Consumption of resources, recyclability
Synthesis greenness	Use of biogenic/waste precursors, benign solvents
Stability/reusability	Lifetime, metal leaching, structural integrity

## Data Availability

Not applicable.

## Nomenclature

C	Concentration of contaminant; mg/L
C_0_	Initial concentration of contaminant; mg/L
k	Reaction rate constant; varies
kr	Intrinsic reaction rate constant; s^−1^
K	Adsorption equilibrium constant; L/mg
t	Irradiation time; min
r	Reaction rate; mg/L∙min
θ	Surface coverage; dimensionless
q	Adsorbed amount; mg/g
Q_t_	Adsorbed amount at time t; mg/g
Q_e_	Adsorbed amount at equilibrium; mg/g
N	Number of viable microbes at time t
N_0_	Initial number of viable microbes
F	Photon flux; mW/cm^2^
D	Diffusion coefficient; m^2^/s
dp	Particle diameter; m
